# Physicians’ attitudes and knowledge concerning antibiotic prescription and resistance: questionnaire development and reliability

**DOI:** 10.1186/s12879-015-1332-y

**Published:** 2016-01-08

**Authors:** António Teixeira Rodrigues, Mónica Ferreira, Fátima Roque, Amílcar Falcão, Elmano Ramalheira, Adolfo Figueiras, Maria Teresa Herdeiro

**Affiliations:** 1 Department of Medical Sciences and Institute for Biomedicine – iBiMED, University of Aveiro, Aveiro, 3810-193 Portugal; 2 Faculty of Pharmacy, University of Coimbra, Coimbra, Portugal; 3 Research Unit for Inland Development, Polytechnic Institute of Guarda, Guarda, Portugal; 4 Centre for Neuroscience and Cell Biology, University of Coimbra, Coimbra, Portugal; 5 Hospital Infante D. Pedro, EPE, Aveiro, Portugal; 6 University of Santiago de Compostela, Santiago de Compostela, Spain; 7 Consortium for Biomedical Research in Epidemiology & Public Health (CIBER en Epidemiología y Salud Pública -CIBERESP), Santiago de Compostela, Spain; 8 CESPU, IINFACTS, Instituto de Investigação e Formação Avançada em Ciências e Tecnologias da Saúde, Gandra, Portugal

**Keywords:** Attitudes, Knowledge, Antibiotic resistance, Questionnaire, Reliability

## Abstract

**Background:**

Understanding physicians’ antibiotic-prescribing behaviour is fundamental when it comes to improving antibiotic use and tackling the growing rates of antimicrobial resistance. The aim of the study was to develop and validate -in terms of face validity, content validity and reliability- an instrument designed to assess the attitudes and knowledge underlying physician antibiotic prescribing.

**Methods:**

The questionnaire development and validation process comprised two different steps, namely: (1) content and face validation, which included a literature review and validation both by physicians and by Portuguese language and clinical psychology experts; and (2) reliability analysis, using the test-retest method, to assess the questionnaire’s internal consistency (Cronbach’s alpha) and reproducibility (intraclass correlation coefficient - ICC). The questionnaire includes 17 items assessing attitudes and knowledge about antibiotic prescribing and resistances and 9 items evaluating the importance of different sources of knowledge. The study was conducted in the catchment area covered by Portugal’s Northern Regional Health Administration and used a convenience sample of 61 primary-care and 50 hospital-care physicians.

**Results:**

Response rate was 64 % (49 % to retest) for primary-care physicians and 66 % (60 % to retest) for hospital-care physicians. Content validity resulted in 9 changes to professional concepts. Face validity assessment resulted in 19 changes to linguistic and interpretative terms. In the case of the reliability analysis, the ICC values indicated a minimum of fair to good reproducibility (ICC > 0.4), and the Cronbach alpha values were satisfactory (α > 0.70).

**Conclusions:**

The questionnaire developed is valid -in terms of face validity, content validity and reliability- for assessing physicians’ attitudes to and knowledge of antibiotic prescribing and resistance, in both hospital and primary-care settings, and could be a very useful tool for characterising physicians’ antibiotic-prescribing behaviour.

**Electronic supplementary material:**

The online version of this article (doi:10.1186/s12879-015-1332-y) contains supplementary material, which is available to authorized users.

## Background

Rates of antimicrobial resistance are growing worldwide, threatening public health and increasing morbidity, mortality and healthcare costs [1–3]. Research suggests that what underlies such high rates of antimicrobial resistance is the misuse of antibiotics in both human and veterinary practices [4–7].

Bearing in mind that antibiotic consumption in southern European countries is higher than that in the north of Europe [8], without obvious benefit to public health [9], it becomes fundamental to understand which factors underlie antibiotic misuse. Considering the pivotal role that physicians play in this process, an in-depth understanding of their attitudes to and knowledge of antibiotic prescribing and antimicrobial resistance is essential when it comes to developing effective interventions and improving antibiotic use [10, 11].

Two recent systematic reviews have highlighted physicians’ attitudes and knowledge as factors affecting physician antibiotic prescribing behaviour [12, 13]; those reviews were based on several qualitative and quantitative studies in different settings; however, any tool have been developed, validated and published which, based on these studies, allow researchers, health professionals and health authorities to measure these specific determinants of antibiotic prescribing.

Questionnaires are well-established tools for collecting data in health sciences and could be a very useful instruments for assessing physicians’ clinical-practice characteristics [14] and understanding their antibiotic-prescribing behaviour.

Several scales have been published for assessing antibiotic misuse and overuse [15]; these scales evaluate the relationship between patients’/parents’ [16] or physicians’ [17] characteristics and antibiotic misuse in the community. According to Alumran et al., however, such scales display weaknesses in development and assessment of validation, which hinder their use in different settings or countries [15]. Measurement of factors underlying physician misprescription of antibiotics should be made using reliable scales because these factors are extremely difficult to identify and assess.

Indeed, several parameters should be assessed to ensure the usefulness of the data collected, so that each question measures what it is intended to measure, all words and sentences are clearly understood, and all participants interpret the questions in the same way [18].

This study sought to develop and validate -in terms of face validity, content validity and reliability- an instrument designed to assess the attitudes and knowledge underlying physician antibiotic prescribing, in order to understand and improve antibiotic use in both hospital- and primary-care settings.

## Methods

A questionnaire was developed with a dual purpose, namely: to assess physicians’ attitudes and knowledge vis-à-vis antibiotic prescribing, antibiotic use and antibiotic resistance, and the usefulness of different sources of knowledge used in clinical practice; and to collect socio-demographic and professional data on the physicians surveyed, including age, gender, medical specialisation, workplace and workflow. The final questionnaire took the form of a two-page long, self-administered document that was easy to complete, not time-consuming, and made up of the following five sections:

- Section 1: instructions to complete the questionnaire;
- Section 2 (“Antibiotics and Resistance”): 17 statements regarding attitudes and knowledge about antibiotic prescribing, antibiotic use and antimicrobial resistance;
- Section 3 (“In the treatment of respiratory infections, how would you rate the usefulness of each of these sources of knowledge?”): 9 statements regarding the importance of various sources of knowledge, which can helps to understand the sources of knowledge underlying antibiotic misprescription;
- Section 4: Socio-demographic and professional data (age, gender, medical specialisation, workplace and workflow);
- Section 5: free to express ideas and views on antibiotics and resistance.

In sections 2 and 3, agreement with statements was measured using a horizontal, continuous, visual analogue scale (VAS), 8 cm long and unnumbered, scored from full disagreement to full agreement.

**Fig. 1:**
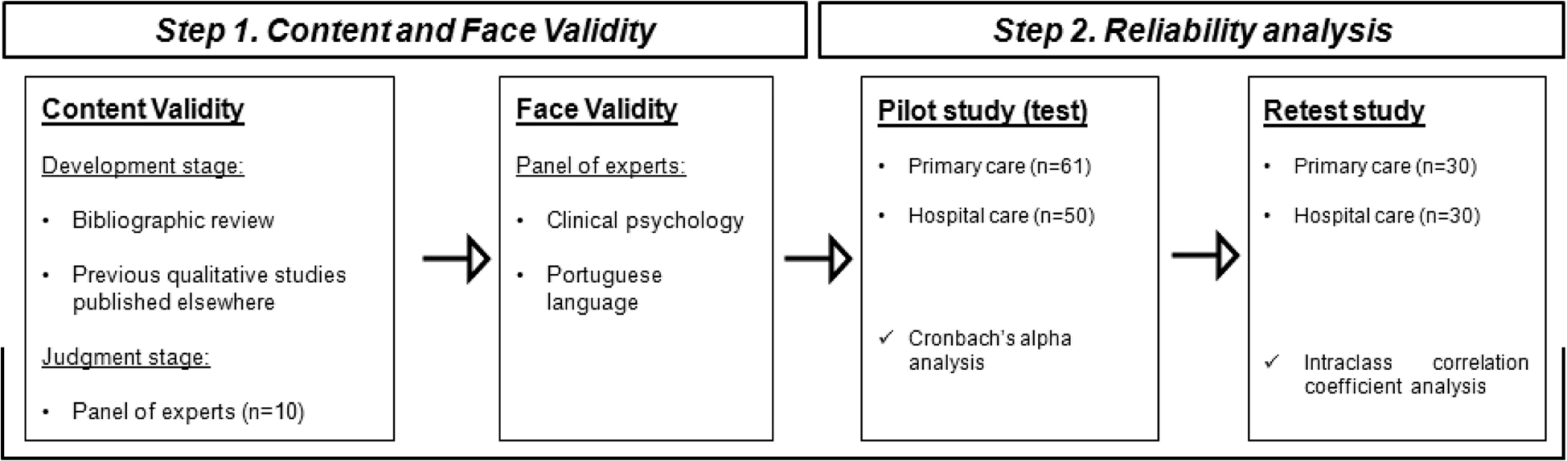
Flow diagram of questionnaire development and validation

Figure 1 depicts the different steps of the study. The study was previously approved by the Northern Regional Health Administration (Permit No. 010484/ 2011), primary-care facility directors and Hospital Administration & Ethics Committee (Permit Date 10/2011). Authorisation from the Portuguese Data Protection Authority (Comissão Nacional de Proteção de Dados/ CNPD) was also obtained (Permit No. 2886/ 2013).

### Step 1. Content and face validity of the questionnaire

Content validity was evaluated in two different stages, in accordance with published guidelines [19]:

(i) The development stage, which included the build-up of the questionnaire and determination of the domain and concepts of the construct of interest. This stage consisted of a literature review [12, 13] and previous studies undertaken by a collaborative group in Spain [20]. It is important to refer that section 2 (17 statements about antibiotics and resistances) resulted from the literature review and concern the most important attitudes identified as influencing antibiotic prescribing. On the other hand, section 3 present the sources of knowledge identified in the literature review as being used by physicians nowadays.
(ii) The judgment stage, in which the professional opinion of experts was evaluated. A convenience sample of two groups of physicians, one consisting of five primary-care physicians and the other of five specialists (2 internists, 2 paediatricians and 1 clinical pathologist) pre-tested the questionnaire and assessed the accuracy, clinical terminology, completeness and meaning of all the statements. An additional panel of two pharmacologists and two pharmacoepidemiologists also evaluated the content validity of the questionnaire.

Face validity, which includes an assessment of the grammar, syntax, organisation, appropriateness and logical sequence of the statements [15], was evaluated by two university professors, one an expert in clinical psychology and the other an expert in the Portuguese language.

### Step 2. Reliability analysis

For an instrument to be valid, it must first be reliable [21]. Reliability refers to the consistency of a test or measurement [22]. To assess questionnaire reliability, two aspects were addressed, i.e., reproducibility and internal consistency.

In September 2013 we used the test-retest method to conduct a pilot test in a so-called NUTS II area (*Nomenclatura das Unidades Territoriais para Fins Estatísticos*/Nomenclature of Territorial Units for Statistics) of Portugal defined by the Northern Regional Health Administration (*Administração Regional de Saúde do Norte*, *I.P*./*ARS*-*N*), using a convenience sample of 61 primary-care and 50 hospital-care physicians. The questionnaire, accompanied by an explanatory letter outlining the study, was personally delivered to the head of each unit who then proceeded to distribute it to all participants who agreed to take part. In line with previous studies [23, 24], an interval of 2 to 4 weeks was allowed to elapse between the first and second administration of the questionnaire.

### Statistical analysis

Agreement between answers was assessed by calculating the intraclass correlation coefficient (ICC), a measure of reproducibility of repeated measures on the same subject [25]. A one-way ICC was chosen because the effect of trials is not cross-checked against subjects (replication study; hence one-way) and the analysis is used to generalise from a trial that used a sample (thus random) [22].

Internal consistency was evaluated using Cronbach’s alpha [26], which is a very useful parameter to describe the extent to which all the items in a test measure the same concept or construct, and it is thus connected to the inter-relatedness of the items within the test [27]. When multiple traits underlie the items on a scale, the “tau equivalent model” assumption is not respected and Cronbach’s alpha underestimates internal consistency [27]. Since Section-2 statements evaluate different concepts (physicians’ attitudes and knowledge), assessing Cronbach’s alpha may be inappropriate according to the assumptions of this parameter. On the other hand, in view of the fact that Section 3 of the questionnaire exclusively evaluates one concept (the utility of different sources of knowledge), Cronbach’s alpha would seem to be a very useful parameter for assessing the internal consistency of this section.

## Results

The response rates for the two groups of physicians studied were as follows: 64 % (*n* = 39) for the first questionnaire administered and 49 % (*n* = 30) for the retest among the 61 primary-care physicians, and 66 % (*n* = 33) and 60 % (*n* = 30) respectively among the 50 hospital-care physicians.

Based on data drawn from Section 4 of the questionnaire, Table 1 gives a breakdown of the socio-demographic and professional characteristics of the physicians included in the study. Notwithstanding the results reported above on the participants’ socio-demographic and professional characteristics, this study did not seek to evaluate the influence of any of these variables.

**Table 1:** Socio-demographic and professional characteristics of the study participants

	Primary-care physicians	Hospital-care physicians
	% (*n* = 30)	% (*n* = 30)
Age (years)		
[25; 45]	57 % (17)	63 % (19)
[46; 65]	43 % (13)	37 % (11)
Gender		
Female	67 % (20)	60 % (18)
Male	33 % (10)	40 % (12)
Medical specialisation		
General practitioner	100 % (30)	3 % (1)
Internal medicine	-	37 % (11)
Paediatrics	-	20 % (6)
Pneumology	-	23 % (7)
Nephrology	-	9 % (3)
Oncology	-	3 % (1)
Cardiology	-	3 % (1)
Activity in		
Public practice	70 % (21)	77 % (23)
Public and private practice	30 % (9)	23 % (7)
Setting		
Hospital care	0 % (0)	77 % (23)
Primary care	73 % (22)	0 % (0)
Both	20 % (6)	23 % (7)
NI^a^	6 % (2)	-
Emergency activity		
Yes	77 % (23)	93 % (28)
No	23 % (7)	7 % (2)

*NI* ^a^ no information available

### Step 1. Content and face validity of the questionnaire

The content-validity judgment stage consisted of the pre-test and professional appraisal of the questionnaire. It was assessed by two groups of physicians who made 9 changes (e.g., in Statement 6, “The use of antibiotics on animals is an important cause of the appearance of new resistance” was amended to read, “The use of antibiotics on animals is an important cause of the appearance of new resistance to pathogenic agents in humans”).

Face-validity assessment resulted in 19 changes to linguistic and interpretative terms in the questionnaire, e.g., Statement 2 was changed from “In a primary-care context, one should wait for the microbiology results to treat an infectious disease” to “In a primary-care context, one should wait for the microbiology results before treating an infectious disease.”

### Step 2. Reliability analysis

Table 2 shows the ICC and Cronbach’s alpha results if any given item is deleted for both groups of physicians included in the study.

**Table 2:** Intraclass correlation coefficients (ICCs) for primary-care and hospital-care physicians

	Primary-care physicians	Hospital-care physicians
Section 2 – Antibiotics and resistance		
Statement	ICC (95 % CI)	ICC (95 % CI)
S 1: Antibiotic resistance is an important Public Health problem in our setting (Ignorance).	0.873 (0.733; 0.940)***	0.711 (0.394; 0.863)***
S 2: In a primary-care context, one should wait for the microbiology results before treating an infectious disease (Ignorance).	0.530 (0.013; 0.776)*	0.751 (0.477; 0.882)***
S 3: Rapid and effective diagnostic techniques are required for diagnosis of infectious diseases (Responsibility of others – Health care System).	0.542 (0.038; 0.782)*	0.590 (0.138; 0.805)**
S 4: The prescription of an antibiotic to a patient does not influence the possible appearance of resistance (Ignorance).	0.432 (−0.193; 0.730)	0.906 (0.801; 0.955)***
S 5: I am convinced that new antibiotics will be developed to solve the problem of resistance (Responsibility of others – Investigation)	0.548 (0.050; 0.785)*	0.922 (0.836; 0.963)***
S 6: The use of antibiotics on animals is an important cause of the appearance of new resistance to pathogenic agents in humans (Responsibility of others)	0.858 (0.703; 0.933)***	0.509 (−0.031; 0.766)*
S 7: In case of doubt, it is preferable to use a wide-spectrum antibiotic to ensure that the patient is cured of an infection (Fear).	0.777 (0.532; 0.894)***	0.926 (0.845; 0.965)***
S 8: I frequently prescribe an antibiotic in situations in which it is impossible for me to conduct a systematic follow-up of the patient (Fear).	0.666 (0.297; 0.841)**	0.835 (0.653; 0.922)***
S 9: In situations of doubt as to whether a disease might be of bacterial aetiology, it is preferable to prescribe an antibiotic (Fear).	0.402 (−0.256; 0.715)	0.859 (0.704; 0.933)***
S 10: I frequently prescribe antibiotics because patients insist on it (Complacency).	0.864 (0.715; 0.935)***	0.429 (−0.200; 0.728)
S 11: I sometimes prescribe antibiotics so that patients continue to trust me (Complacency).	0.857 (0.700; 0.932)***	0.855 (0.695; 0.931)***
S 12: I sometimes prescribe antibiotics, even when I know that they are not indicated because I do not have the time to explain to the patient the reason why they are not called for (Indifference).	0.860 (0.705; 0.923)***	0.946 (0.887; 0.974)***
S 13: If a patient feels that he/she needs antibiotics, he/she will manage to obtain them at the pharmacy without a prescription, even when they have not been prescribed (Responsibility of others – Other Professionals).	0.822 (0.625; 0.915)***	0.753 (0.481; 0.882)***
S 14: Two of the main causes of the appearance of antibiotic resistance are patient self-medication and antibiotic misuse (Responsibility of others – Patients).	0.767 (0.510; 0.889)***	0.468 (−0.117; 0.747)
S 15: Dispensing antibiotics without a prescription should be more closely controlled (Responsibility of others – Health care System).	0.683 (0.325; 0.851)**	0.692 (0.353; 0.853)**
S 16: In a primary-care context, amoxicillin is useful for treating most respiratory infections (Ignorance).	0.470 (−0.074; 0.748)	0.745 (0.465; 0.879)***
S 17: The phenomenon of resistance to antibiotics is mainly a problem in hospital settings (Responsibility of others – Other Professionals).	0.690 (0.348. 0.852)**	0.706 (0.382; 0.860)**
Section 3 – In the treatment of respiratory tract infections, how would you rate the usefulness of each of these sources of knowledge?		
Statement	ICC (95 % CI)	ICC (95 % CI)
S 1’: Clinical practice guidelines.	0.846 (0.676; 0.927)***	0.562 (0.079; 0.791)*
S 2’: Documentation furnished by the Pharmaceutical Industry.	0.579 (0.116; 0.800)*	0.746 (0.467; 0.879)***
S 3’: Courses held by the Pharmaceutical Industry.	0.519 (−0.011; 0.771)*	0.734 (0.441; 0.873)***
S 4’: Information furnished by Medical Information Officers.	0.851 (0.687; 0.929)***	0.753 (0.481; 0.883)***
S 5’: Previous clinical experience.	0.715 (0.401; 0.864)***	0.714 (0.399; 0.864)**
S 6’: Continuing Education Courses.	0.708 (0.387; 0.861)***	0.797 (0.574; 0.904)***
S 7’: Others, e.g., contribution of specialists (microbiologists, infectious disease specialists, etc.).	0.948 (0.890; 0.975)***	0.595 (0.148; 0.807)**
S 8’: Contribution of peers (of the same specialisation).	0.764 (0.505; 0.888)***	0.655 (0.275; 0.836)**
S 9’: Data collected via the Internet.	0.723 (0.419; 0.868)**	0.762 (0.500; 0.887)***

*p < 0.05; **p < 0.01; ***p < 0.001

The Cronbach alpha values yielded for Section 3 of the questionnaire were satisfactory (α > 0.70) [28] for both groups of physicians studied (α_Hospital-care physicians_: 0.783; α_Primary-care physicians_: 0.770).

Applying the definition proposed by *Roster B*. [25], ICC values smaller than 0.4 can be taken to indicate poor reproducibility, those ranging from 0.4 to 0.75 to indicate fair to good reproducibility, and those above 0.75 to indicate excellent reproducibility. On this basis, all statements displayed a minimum of fair to good reproducibility in both groups of physicians studied and in both sections of the questionnaire. Eight (Section 2) and four (Section 3) statements by primary-care physicians and ten (Section 2) and two (Section 3) statements by hospital-care physicians study yielded excellent ICC values.

When comparing the ICCs obtained for the two groups of physicians, considerable differences were found with respect to six statements in Section 2 (S4, S6, S9, S10, S14 and S16) and four statements in Section 3 (S1’, S2’, S3’ and S7’).

## Discussion

Assessing knowledge and attitudes that guide antibiotic prescribing is an essential step when it comes to counteracting the problem of antimicrobial resistance. Several scales have been developed to measure the factors associated with antibiotic misuse world-wide [29–31] but most of them have not been fully validated [15]. This study reports on the development process and reliability evaluation of a questionnaire (Additional file 1) designed to assess physicians’ knowledge and attitudes concerning antibiotic use. The results yielded show that the questionnaire: (i) has content validity, face validity and is reliable in terms of internal consistency and reproducibility over time; and, (ii) could be applied to physicians working in both primary-care and hospital-care settings.

In terms of public health issues and related policies, this questionnaire for assessing physicians’ knowledge and attitudes offers several advantages over other published instruments, in that: (i) it allows for assessment, identification and description of attitudes and knowledge regarding antibiotic prescribing and resistance among hospital- and primary-care physicians simultaneously, and this in turn allows for comparison between the two groups of professionals; and, (ii) it uses a VAS which is a highly interesting scale for assessing small differences between physicians and between groups of physicians.

Indeed, considering the growing rates of antimicrobial resistance and decreasing effectiveness of antibiotics, antibiotic misuse is a major public health issue around the world, to the point where it is threatening a return to the pre-antibiotic era. Bibliographic reviews [12, 13] of physician antibiotic-prescribing behaviour have highlighted intrinsic factors, such as their socio-demographic characteristics, attitudes and diagnostic uncertainty, as being at the root of antibiotic misprescription. The literature also points the collegial advice and the logistics of microbiology test results as factors affecting antibiotic prescribing [32]. This questionnaire now enables factors -such as attitudes- related with antibiotic misuse to be identified.

Several interventions have been implemented around the world, targeting at improving antibiotic prescribing among primary-care [33] and hospital-care [34] professionals. Considering that these interventions should reflect the characteristics and barriers present in the setting where they are to be implemented, this questionnaire is an adequate instrument for assessing the factors affecting physician-prescribing behaviour, an essential pre-requisite for developing effective educational interventions and improving antibiotic use. When we aim to assess and improve attitudes influence on antibiotic prescribing, we can also use this questionnaire to evaluate the intervention effect (pre- vs post-assessment) on these determinants. Finally, use this questionnaire in different countries/ settings will also allows to compare the influence of this factors in different realities, which could help to understand different rates of antibiotic prescribing quality indicators in different countries.

### Questionnaire development

Visual analogue scales are well-established instruments for collecting data in several different areas [35–37] and numerous studies have supported their usefulness, validity and reliability, when compared to Likert scales [38, 39]. While both scales capture similar information, a VAS assesses and enables the quantification of subjective phenomena, and might be able to discern subtle differences because of its greater range of possible scores [40, 41].

Face validity, as a form of validity that refers to a subjective assessment [42], and content validity, as a measure of the comprehensiveness and representativeness of a scale’s content [19], were assessed and guaranteed by the panels of experts, the literature review [12] and a qualitative study conducted by a collaborative group [20].

With regard to the methodology used in the pilot study (test-retest), the main problem lies in the potential for learning or recall effects that can affect the test [43]. The 2-week interval was fundamental to ensure that the time was [24]: (i) long enough to avoid carry-over effects due to memory; and, (ii) short enough to avoid changes in physicians’ knowledge or attitudes concerning antibiotic prescribing and antimicrobial resistance.

The high Cronbach’s alpha values obtained in Section 3 of the questionnaire (Primary-care physicians: 0.770; Hospital-care physicians: 0.783) revealed the internal consistency of the statements in this section, and we feel that this is linked to the objectivity of these statements, which exclusively evaluate the utility of different sources of knowledge.

A comparison of the results obtained for the two groups of physicians could be very useful for assessing the appropriateness of the questionnaire and its applicability in each setting. The usefulness of the questionnaire was not the same for both groups of physicians. We feel, however, that these differences were related to the specific characteristics of each group, e.g., the differences found in Statement 9 of section 2 (“In situations of doubt as to whether a disease might be of bacterial aetiology, it is preferable to prescribe an antibiotic.”) could well be related to the availability in a hospital setting of microbiological tests that are not to be found in primary-care facilities. As regards the differences seen in the Section 3 of the questionnaire, the fair ICCs obtained for statements about the information and courses provided by the pharmaceutical industry (S2 and S3), could be related to primary-care physicians’ greater exposure to sales representatives, something that is in line with the reported influence of pharmaceutical companies in primary-care physician training [44].

This study’s main limitation lay in the size and selection of the sample. Even so, 30 participants in each group of physicians would seem to be a reasonable number for a pilot study where the purpose is preliminary survey or scale development [45]. However, this sample size doesn’t allow to confirm the construct validity using exploratory factor analysis, which should be performed in future research with larger samples. While sample selection by a convenience method is the most widely-used process in validation studies, it nevertheless constituted our study’s main limitation [46]. TTo evaluate the construct validity of the questionnaire, a larger sample size is needed; however, to confirm the structure presented, future research should also evaluate this using exploratory factor analysis.

## Conclusion

Bearing in mind that antibiotic resistance is one of the main public health problems world-wide, this questionnaire would seem to be a very useful tool for collecting data on physicians’ attitudes to and knowledge of antibiotic prescribing and resistance, which is one of the most important factors underlying this global concern. Hence, this questionnaire should be used to assess factors underlying physician-prescribing behaviour in hospital and primary care, along with other possible variables, such as socio-demographic or professional-practice variables (medical specialisation, workplace and workflow). We believe that the use of this questionnaire could prove of the utmost importance when it comes to identifying factors underlying antibiotic prescription and antimicrobial resistance in both hospital and primary care.

Questionnaire development would seem to be essential for improving the likelihood of success in achieving the main study objective, which, in this case, is to identify factors underlying antibiotic prescription and antimicrobial resistance. This study presents a very comprehensive questionnaire which could be used on both primary- and hospital-care physicians.

## Additional file

Additional file 1:
**Description of data: Questionnaire evaluated in the study.** (PDF 312 kb)

## References

[1] World Health Organization (2012). Antibiotic resistance.

[2] Howard DH, Scott RD (2005). The economic burden of drug resistance. Clin Infect Dis.

[3] Kollef MH (2003). The importance of appropriate initial antibiotic therapy for hospital-acquired infections. Am J Med.

[4] Albrich WC, Monnet DL, Harbarth S (2004). Antibiotic selection pressure and resistance in Streptococcus pneumoniae and Streptococcus pyogenes. Emerg Infect Dis.

[5] Wise R, Hart T, Cars O, Streulens M, Helmuth R, Huovinen P (1998). Antimicrobial resistance. Is a major threat to public health. BMJ.

[6] Goossens H, Ferech M, Vander Stichele R, Elseviers M (2005). Outpatient antibiotic use in Europe and association with resistance: a cross-national database study. Lancet.

[7] Tseng R (1985). An audit of antibiotic prescribing in general practice using sore throats as a tracer for quality control. Public Health.

[8] European Centre for Disease Prevention and Control (2012). Antimicrobial resistance surveillance in Europe 2011. Annual Report of the European Antimicrobial Resistance Surveillance Network (EARS-Net).

[9] Butler CC, Hood K, Verheij T, Little P, Melbye H, Nuttall J (2009). Variation in antibiotic prescribing and its impact on recovery in patients with acute cough in primary care: prospective study in 13 countries. BMJ.

[10] Tonkin-Crine S, Yardley L, Little P (2011). Antibiotic prescribing for acute respiratory tract infections in primary care: a systematic review and meta-ethnography. J Antimicrob Chemother.

[11] Chlabicz S, Pytel-Krolczuk B (2008). Antibiotic treatment for respiratory tract infections in Polish primary care facilities: is it time to change national guidelines or doctor prescribing behaviour?. J Eval Clin Pract.

[12] Teixeira Rodrigues A, Roque F, Falcao A, Figueiras A, Herdeiro MT (2013). Understanding physician antibiotic prescribing behaviour: a systematic review of qualitative studies. Int J Antimicrob Agents.

[13] Lopez-Vazquez P, Vazquez-Lago JM, Figueiras A (2012). Misprescription of antibiotics in primary care: a critical systematic review of its determinants. J Eval Clin Pract.

[14] Den Heijer M, van Asperen CJ, Harris H, Nippert I, Schmidtke J, Bouhnik AD (2013). International variation in physicians’ attitudes towards prophylactic mastectomy - comparison between France, Germany, the Netherlands and the United Kingdom. Eur J Cancer.

[15] Alumran A, Hou XY, Hurst C (2012). Validity and reliability of instruments designed to measure factors influencing the overuse of antibiotics. J Infect Public Health.

[16] Belongia EA, Naimi TS, Gale CM, Besser RE (2002). Antibiotic use and upper respiratory infections: a survey of knowledge, attitudes, and experience in Wisconsin and Minnesota. Prev Med.

[17] Coenen S, Michiels B, Van Royen P, Van der Auwera JC, Denekens J (2002). Antibiotics for coughing in general practice: a questionnaire study to quantify and condense the reasons for prescribing. BMC Fam Pract.

[18] Taylor-Powell E. Pilot test your questionnaire. University of Wisconsin-Extension, Cooperative Extension. 2008. Available: https://www.team-psa.com/brfss/2012/pres/K_Trepanier_questionnaire.pdf.

[19] Yaghmaie F (2003). Content validity and its estimation. J Med Educ.

[20] Vazquez-Lago JM, Lopez-Vazquez P, Lopez-Duran A, Taracido-Trunk M, Figueiras A (2012). Attitudes of primary care physicians to the prescribing of antibiotics and antimicrobial resistance: a qualitative study from Spain. Fam Pract.

[21] Szklo M, Nieto J (2003). Epidemiologia intermedia: conceptos y aplicaciones.

[22] Weir JP (2005). Quantifying test-retest reliability using the intraclass correlation coefficient and the SEM. J Strength Cond Res.

[23] dos Santos Pernas SI, Herdeiro MT, Lopez-Gonzalez E (2012). da Cruz e Silva OA, Figueiras A. Attitudes of Portuguese health professionals toward adverse drug reaction reporting. Int J Clin Pharm.

[24] Marx RG, Menezes A, Horovitz L, Jones EC, Warren RF (2003). A comparison of two time intervals for test-retest reliability of health status instruments. J Clin Epidemiol.

[25] Rosner B (2006). Fundamentals of Biostatistics.

[26] Cronbach LJ (1951). Coefficient alpha and the internal structure of tests. Psychometrika.

[27] Tavakol M, Dennick R (2011). Making sense of Cronbach’ alpha. Int J Med Educ.

[28] Martin Bland J, Altman DG (1997). Statistics notes: Cronbach’s alpha. BMJ.

[29] Liabsuetrakul T, Chongsuvivatwong V, Lumbiganon P, Lindmark G (2003). Obstetricians’ attitudes, subjective norms, perceived controls, and intentions on antibiotic prophylaxis in caesarean section. Soc Sci Med.

[30] Gould IM, Mackenzie FM, Shepherd L (2007). Attitudes to antibiotic prescribing, resistance and bacteriology investigations amongst practitioners and patients in the Grampian region of Scotland. Eur J Gen Pract.

[31] Weiss MC, Deave T, Peters TJ, Salisbury C (2004). Perceptions of patient expectation for an antibiotic: a comparison of walk-in centre nurses and GPs. Fam Pract.

[32] Skodvin B, Aase K, Charani E, Holmes A, Smith I (2015). An antimicrobial stewardship program initiative: a qualitative study on prescribing practices among hospital doctors. Antimicrob Resistance Infect Control.

[33] Arnold SR, Straus SE (2005). Interventions to improve antibiotic prescribing practices in ambulatory care. Cochrane Database Syst Rev.

[34] Davey P, Brown E, Fenelon L, Finch R, Gould I, Hartman G (2005). Interventions to improve antibiotic prescribing practices for hospital inpatients. Cochrane Database Syst Rev.

[35] Miller MD, Ferris DG (1993). Measurement of subjective phenomena in primary care research: the Visual Analogue Scale. Fam Pract Res J.

[36] Herdeiro MT, Figueiras A, Polonia J, Gestal-Otero JJ (2005). Physicians’ attitudes and adverse drug reaction reporting : a case–control study in Portugal. Drug Saf.

[37] Figueiras A, Tato F, Fontainas J, Gestal-Otero JJ (1999). Influence of physicians’ attitudes on reporting adverse drug events: a case–control study. Med Care.

[38] Pfennings L, Cohen L, van der Ploeg H (1995). Preconditions for sensitivity in measuring change: visual analogue scales compared to rating scales in a Likert format. Psychol Rep.

[39] Grant S, Aitchison T, Henderson E, Christie J, Zare S, McMurray J (1999). A comparison of the reproducibility and the sensitivity to change of visual analogue scales, Borg scales, and Likert scales in normal subjects during submaximal exercise. Chest.

[40] Celenza A, Rogers IR (2011). Comparison of visual analogue and Likert scales in evaluation of an emergency department bedside teaching programme. Emerg Med Australas.

[41] Foley DK (2008). Development of a visual analogue scale to measure curriculum outcomes. J Nurs Educ.

[42] DeVon HA, Block ME, Moyle-Wright P, Ernst DM, Hayden SJ, Lazzara DJ (2007). A psychometric toolbox for testing validity and reliability. J Nurs Scholarsh.

[43] Allen MJ, Yen WM (1979). Introduction to measure theory.

[44] Caamano F, Figueiras A, Gestal-Otero JJ (2002). Influence of commercial information on prescription quantity in primary care. Eur J Public Health.

[45] Johanson GA, Brooks GP (2009). Initial Scale Development: Sample Size for Pilot Studies. Educ Psychol Meas.

[46] Bonita R, Beaglehole R, Kjellstrom T (2006). Basic epidemiology.

