# Supplementary material for: Physicians’ attitudes and knowledge concerning antibiotic prescription and resistance: questionnaire development and reliability
Source: BMC Infect Dis. 2016 Jan 8;16:7. doi: 10.1186/s12879-015-1332-y (PMC4705586; doi:10.1186/s12879-015-1332-y)
Supplement: Additional file 1: — Description of data: Questionnaire evaluated in the study. (PDF 312 kb) [file 12879_2015_1332_MOESM1_ESM.pdf]

universidade de aveiro

QUESTIONNAIRE Nº:_____

FILLING INSTRUTIONS	totally disagree — totally agree
In the left column are questions that will be the subject of your evaluation and in the right column there is a **gradual scale** where you should mark with a cross the place where, accordingly to your opinion represents your agreement with the text comment. If you are totally in disagreement, you should place a cross at the left end, and as your agreement increases you should move the cross to the right.	|X———————————| 0 100% |—————X—————| |———————————X|

ABOUT ANTIBIOTICS AND RESISTANCES

	Totally disagree — totally agree
1. Antibiotic resistance is an important Public Health problem in our setting.	|———————————|
2. In a primary-care context, one should wait for the microbiology results before treating an infectious disease.	|———————————|
3. Rapid and effective diagnostic techniques are required for diagnosis of infectious diseases.	|———————————|
4. The prescription of an antibiotic to a patient does not influence the possible appearance of resistance.	|———————————|
5. I am convinced that new antibiotics will be developed to solve the problem of resistance.	|———————————|
6. The use of antibiotics on animals is an important cause of the appearance of new resistance to pathogenic agents in humans.	|———————————|
7. In case of doubt, it is preferable to use a wide-spectrum antibiotic to ensure that the patient is cured of an infection.	|———————————|
8. I frequently prescribe an antibiotic in situations in which it is impossible for me to conduct a systematic follow-up of the patient.	|———————————|
9. In situations of doubt as to whether a disease might be of bacterial aetiology, it is preferable to prescribe an antibiotic.	|———————————|
10. I frequently prescribe antibiotics because patients insist on it.	|———————————|
11. I sometimes prescribe antibiotics so that patients continue to trust me.	|———————————|
12. I sometimes prescribe antibiotics, even when I know that they are not indicated because I do not have the time to explain to the patient the reason why they are not called for.	|———————————|
13. If a patient feels that he/she needs antibiotics, he/she will manage to obtain them at the pharmacy without a prescription, even when they have not been prescribed.	|———————————|
14. Two of the main causes of the appearance of antibiotic resistance are patient self-medication and antibiotic misuse.	|———————————|
15. Dispensing antibiotics without a prescription should be more closely controlled.	|———————————|
16. In a primary-care context, amoxicillin is useful for treating most respiratory infections.	|———————————|
17. The phenomenon of resistance to antibiotics is mainly a problem in hospital settings.	|———————————|

universidade de aveiro

IN THE TREATMENT OF RESPIRATORY TRACT INFECTIONS, HOW WOULD YOU RATE THE USEFULLNESS OF EACH OF THESE SOURCES OF KNOWLEDGE?:

	totally disagree ——— totally agree
Clinical practice guidelines.	|———|
Documentation furnished by the Pharmaceutical Industry.	|———|
Courses held by the Pharmaceutical Industry.	|———|
Information furnished by Medical Information Officers.	|———|
Previous clinical experience.	|———|
Continuing Education Courses.	|———|
Others, e.g., contribution of specialists (microbiologists, infectious disease specialists, etc.).	|———|
Contribution of peers (of the same specialisation).	|———|
Data collected via the Internet.	|———|

Some questions about sociodemographic data and about your clinical practice

How old are you? _____ years

Gender: F ☐ M ☐

What is your medical specialization?

____

What type of activity?

Public practice ☐

Private practice ☐

Both ☐

In which workplace?

Hospital care ☐

Primary care ☐

Both ☐

Do you work at the emergency service?

Yes ☐

No ☐

Approximately, what is the number of patients seen per day? _____ patients

Approximately, what is the number of patients seen per day at the emergency service? _____ patients

Approximately, how much time do you need to attend one patient?

_____ minutes

DO YOU HAVE SOME SUGESTIONS ABOUT ANTIBIOTIC USE AND RESISTANCES?

THANK YOU VERY MUCH FOR YOUR COOPERATION!
